# Improving transparency in clinical trial reporting

**DOI:** 10.1371/journal.pmed.1004588

**Published:** 2025-04-28

**Authors:** Alison Farrell, On Behalf of the PLOS Medicine Editors

**Affiliations:** Public Library of Science, San Francisco, California, United States of America

## Abstract

*The power to interpret the results of randomised clinical trial results relies on transparent reporting of the study design, protocol, methods and analyses. Without such clarity, the benefits of the findings, to both healthcare, policy and research, cannot be realized in full. The publication of the updated CONSORT 2025 and SPIRIT 2025 statements for reporting of randomised clinical trials and protocols, respectively, offers the opportunity to reflect on the power that transparent reporting of clinical trial design and data offers to improve the quality of trials and outcomes*.

The Consolidated Standards of Reporting Trials (CONSORT) statement aims to improve the accuracy and clarity of clinical trial reporting by delineating a minimum number of essential components needed in a primary report to thoroughly evaluate a randomised trial. Designed as an itemized checklist and accompanying flow diagram of participant involvement, with explanatory documents for users articulating the rationale for each checklist item, the statement provides a concise resource of where to access a completed trial’s key details. In this third update to the original 1996 statement, CONSORT 2025 [1] reflects the increasing appreciation of the importance of not only transparent trial reporting, but also of accessibility of study information.

As a complementary set of guidelines, the Standard Protocol Items: Recommendations for Interventional Trials (SPIRIT) statement seeks to increase the comprehensiveness of clinical trial protocol reporting, and was updated alongside CONSORT in view of their aligned goals, and to create consistent guidance. The SPIRIT 2025 statement [2] comprises a conceptually overlapping set of checklist items to those of CONSORT 2025, accompanied by an illustrative diagram, focusing on the completeness of trial protocols to facilitate trial replication, reduce the frequency of protocol amendments and provide accountability for trial design, conduct and data dissemination. The updated CONSORT AND SPIRIT statements are being published simultaneously by The BMJ, JAMA, The Lancet, Nature Medicine, and PLOS Medicine.

These statements are designed as living documents, to be updated as needed to reflect advances in clinical research, methodologies and the needs of participant communities. New additions to both 2025 statements include a section on open science that clarifies the trial registration, the statistical analysis plan and data availability, as well as funding sources and potential conflicts of interest. Such data sharing is vital to both evaluation of trial findings and facilitating their follow-up, yet it remains an unresolved barrier in clinical research, with too much data siloed by restrictive access policies or unavailable altogether [3]. Moving open science priorities to the top of the checklists and thereby underscoring their importance, may, with oversight from journals and reviewers, accelerate and broaden constructive access to de-identified participant and methodological data.

The coordinated revision of the CONSORT and SPIRIT statements speaks to their intended utility over the life course of a trial, for both new and seasoned clinical investigators. While the CONSORT checklist accompanies a report typically drafted after the completion of the full trial, the SPIRIT statement aims to “promote transparency…of what is planned”, with the hope that it would be used as guidance during the planning stage of a trial, before submission of a protocol to an institutional review board.

And there lies the challenge—both checklists are likely completed after trial design and execution, and therefore the recommendations fail to be taken into consideration during early planning stages. Moreover, checklist items can be misinterpreted or not reported in full, resulting in insufficient information in either the trial report or protocol to understand how data were handled and the trial conducted. These omissions undermine a key goal of these checklists—to not only improve reporting but also the generation of reliable and reproducible trial results. While the authors of the statements stress that completion of these checklists should not be interpreted as a measure of the quality of a clinical trial design or its primary report, comprehensive consideration of the crucial reporting elements in the trial design phase could reduce biased representation of study findings and increase their value to both researchers and patients.

Considered in full, the checklist items guide researchers to justify not only the analytical methodology supporting their claims, but importantly why and how a trial was conducted and whether the target patient population or public was involved at any stage. As patient and public involvement is indispensable to identifying healthcare gaps and achieving health impacts of clinical interventions [4], raising awareness of the potential for patient and public participation at early stages can lead to more robust gains at later clinical stages.

In support of open and transparent reporting of clinical trials, PLOS Medicine requires the submission of the study protocol, completed CONSORT checklist and flowchart for internal and external assessment of randomised clinical trials. We are now adopting the updated CONSORT 2025 guidelines for trial reporting and encourage authors to simultaneously include a completed SPIRIT 2025 checklist at the time of full manuscript submission. As medical journals may increasingly require submission of both completed checklist documents for clinical trials, with an expectation that primary reports and protocols contain the requested information in full, we advocate for proactive support of reporting accountability by authors and reviewers. Adherence to the guidance set out in the two statements has the potential to improve study reproducibility, accessibility and, ultimately, advances in health.
